# Time to rethink the systematic review catechism? Moving from ‘what works’ to ‘what happens’

**DOI:** 10.1186/s13643-015-0027-1

**Published:** 2015-03-28

**Authors:** Mark Petticrew

**Affiliations:** Faculty of Public Health and Policy, London School of Hygiene and Tropical Medicine, 15-17 Tavistock Place, London, WC1H 9SH UK

**Keywords:** Systematic reviews, Evidence, Complex interventions

## Abstract

Systematic review methods are developing rapidly, and most researchers would recognise their key methodological aspects, such as a closely focussed question, a comprehensive search, and a focus on synthesising ‘stronger’ rather than ‘weaker’ evidence. However, it may be helpful to question some of these underlying principles, because while they work well for simpler review questions, they may result in overly narrow approaches to more complex questions and interventions. This commentary discusses some core principles of systematic reviews, and how they may require further rethinking, particularly as reviewers turn their attention to increasingly complex issues, where a Bayesian perspective on evidence synthesis, which would aim to assemble evidence - of different types, if necessary - in order to inform decisions’, may be more productive than the ‘traditional’ systematic review model. Among areas identified for future research are the examination of publication bias in qualitative research; research on the efficiency and potential biases of comprehensive searches in different disciplines; and the use of Bayesian methods in evidence synthesis. The incorporation of a systems perspective into systematic reviews is also an area which needs rapid development.

## Background

Systematic reviews are a rapidly maturing technology, and the recent celebrations of 20 years of the Cochrane Collaboration have prompted much taking stock of achievements and some discussion of possible future directions for evidence synthesis methods. The 40th ‘birthday’ of Gene Glass’ seminal meta-analysis on the effectiveness of psychotherapy may prompt further methodological reflection [1]. This paper reflects further on how key aspects of current systematic review practice are either in need of change or are already changing.

## Main text

Chandler and Hopewell [2] have described how evidence synthesis methods have developed rapidly in the past two decades [2], and modern systematic reviews are increasingly characterised by a willingness to incorporate a wider range of study designs [3], to embrace more complex review questions [4,5], and in recent years a willingness to admit qualitative research into systematic reviews, in order to investigate the meaning and acceptability of interventions and to illuminate intervention mechanisms [6]. This evolution in methods has been in train both within and outside the Cochrane Collaboration for some time. In 2006, Dixon-Woods and others [7] described a wide range of positivist and interpretative approaches to evidence synthesis, other than the ‘traditional’ systematic review [8,9], and more recently, realist and meta-narrative reviews have arisen and have developed their own methods and standards for conducting reviews [10-12].

Of course, not all attempts to develop review methods have been embraced rapidly, and Hannes *et al*. [13] have characterised the process of gaining acceptance of qualitative research as ‘mixing progress with rejection’ [13]. However, although the methodological history of systematic reviews is being one of flux and evolution, certain core principles still remain, such as the need for a clear question, the need for transparency of methods, and the use of wide-ranging, comprehensive searches to reduce the effects of publication bias.

Given this constant methodological development, and the appearance of newer review methods, it is legitimate to ask whether there are ‘core’ features of systematic review methods which might be challenged, or which might need to evolve further. This paper identifies a range of potential inter-related challenges to current systematic review practice which seem to merit further consideration.

### 1. Do systematic reviews really need a focused question? Testing hypotheses about ‘what works’ versus answering questions about ‘what happens’

The need for a clearly specified review question, often with a focus on effectiveness, has often been emphasised [14]. The first BMJ book on systematic reviews went even further, noting that systematic reviews are hypothesis-testing mechanisms [15]. The Cochrane Collaboration logo is itself an example of this, with its forest plot illustrating the individual and combined results of seven trials assessing the effectiveness of corticosteroids for premature birth. Answering such closely specified research questions remains a key role for systematic reviews.

The challenge here is that testing hypotheses about whether particular interventions work or not is not the only purpose of systematic reviews. More importantly, when one moves to reviews of more complex, socially embedded interventions, hypothesis-testing is not only difficult, it may not even be desirable. This is because evaluating complex social interventions purely in terms of whether they ‘work’ or ‘do not work’ can be simplistic and misleading. Instead, systematic reviews in these circumstances probably should not aim to make such an overarching, blanket statement, with the implication that the intervention works in all circumstances, but instead should aim to assemble a range of examples of what happened when that intervention was implemented in different contexts. So, instead of using systematic reviews to separate the social world into ‘things that work’ and ‘things that don’t work’, the goal of systematic reviews of complex interventions should be to answer a broader question: *‘What has happened previously when this intervention been implemented across a range of contexts, populations and subpopulations, and how have those effects come about?*’ The results of such an analysis can then be used to inform a decision about how an intervention is likely to behave in other settings. It may be worth noting here that a ‘broader’ question like this is not an ‘unfocussed’ question. It is simply focused on a different question.

This moves us away from systematic reviews which answer simple questions about the effectiveness of complex phenomena. For many interventions, such reviews are undoubtedly the most appropriate, rigorous and scientifically defensible approach to synthesising evidence. However, for many complex social interventions, this is not the case. Asking the simple question ‘does it work?’ about highly complex social change processes, where evidence is often sparse and heterogenous, is often meaningless and usually unanswerable.

Blanket statements about effectiveness are at best a simple starting point for a more detailed investigation of the chain of events which flow from the introduction of an intervention in a complex system. More often in such cases, the most useful synthesis may be one which puts together jigsaws of evidence addressing different aspects of the review question (similar to what has been called a ‘configurative’ approach to reviewing) [16,17]. This is actually consistent with Cochrane handbook guidance on why we do reviews:

‘*… reviews can be conducted in an effort to resolve conflicting evidence, to address questions where clinical practice is uncertain, to explore variations in practice, to confirm the appropriateness of current practice or to highlight a need for future research. The overarching aim of Cochrane reviews should be to summarize and help people to understand the evidence…*’ [18].

In summary, changes in complex systems cannot be understood by simply asking whether they ‘work’, and systematically reviewing studies which ask the same narrow question does not get us any closer to a useful answer.

### 2. Should systematic reviews really avoid duplication?

Good practice in systematic reviewing suggests that one should start by identifying any previous systematic reviews and avoiding duplication. This is a core principle of the Cochrane Collaboration: ‘*Avoiding duplication by good management and coordination to maximize economy of effort*’ [18].

However, quite often the existence of a previous systematic review does not render subsequent reviews unnecessary. One reason for this is that replication is in the nature of science, and systematic reviews remain a human endeavour in which choice and judgement play a part. It is therefore important to replicate selected reviews. Moher makes this point explicitly and differentiates between appropriate and inappropriate duplication: ‘*Not all duplication is bad. Indeed, replication is essential and has uncovered some unfortunate behaviour by scientists clinicians - and other decision makers - can be more confident in the consistency, or lack thereof, of initially reported results*’ [19]. The development of the PROSPERO database should also help reduce inadvertent duplication [20].

Revisiting previous reviews may also be valuable because systematic review methods, and perhaps users’ interests, have changed over the past 20 or so years. One area where this may be legitimate is in the field of complexity, where reviewing the evidence from a systems perspective as opposed to a hypothesis-testing perspective may provide a fuller picture of ‘how’ an intervention works [21]. So, in addition to the above reasons for judicious replication, there may well be a case for reworking a review in the light of new methods and new thinking.

### 3. ‘More’ may not be ‘better’: do literature searches really need to be comprehensive?

Systematic reviewers conduct comprehensive literature searches in order to reduce the risk of missing key studies and to minimise publication bias. The AMSTAR checklist encourages this by asking ‘Was a comprehensive literature search performed?’ [22].

However, there are alternatives to conducting large-scale, scorched-earth searches in the quest for comprehensiveness. Lorenc, Gough and others have discussed the potential of purposive searching and saturation, in which individual studies are sought and included on the basis that they can add conceptually to the review, as opposed to attempting to be comprehensive [16,23]. This may be more applicable to reviews of qualitative research, and the feasibility of such an approach remains to be determined [23]. However, the concept of saturation is also worth considering in relation to quantitative reviews, where there are usually rapidly diminishing returns from large-scale searches. It such cases, the value of the literature search often lies more in deflecting future criticisms of lack of assiduousness in searching, rather than in any real anticipation of finding evidence which would overturn the review’s conclusions.

Even if additional studies are found, new information may make little useful contribution. In a meta-analysis, additional studies can add useful quantitative information because they influence the size of the confidence intervals around the summary effect size. However, in the absence of a meta-analysis, this obviously is not the case. Searching for additional studies may in principle still be useful as it reduces the risk of publication bias, but there may be more efficient ways to do this. One approach might involve regularly ‘taking stock’ of the studies being found, and putting in place stopping rules [4]. In the more distant future, perhaps some more formal cumulative value of information (VOI) analysis may tell us when to safely stop searching [24,25]. Prioritising database searches so that the potentially more productive databases are searched first is also important [25].

Undoubtedly what Lefebvre calls ‘the perennial question of when is enough is enough’ will become ever more important as reviews become more complex, and information retrieval technologies become more efficient. Booth [25] compared a range of possible methods for operationalizing this cost/value decision in deciding when to stop searching, and this remains a priority for future methodological research [25].

Admittedly, there may be risks in abandoning comprehensiveness as a goal - in particular, bias may be introduced if the search ceases when positive conclusions are reached. The concept of saturation may also be difficult to apply when there is little research evidence to begin with (though one might then question whether a systematic review is a good use of resources). However, comprehensiveness is not risk-free either. Egger and colleagues have found that trials that are difficult to locate tend to be smaller and of lower methodological quality than trials that are easily accessible [26]. They also referred to ‘*the worrying possibility that rather than preventing bias through extensive literature searches, bias could be introduced by including trials of low methodological quality*’ [26].

In short, reviewers aim for comprehensiveness for several reasons, including (in the case of meta-analysis) to increase the precision of our summary estimate and to reduce publication bias. However, for narrative reviews, the concept of precision is less relevant; instead, we search for studies to reduce uncertainty about effects, a much broader concept. Uncertainty may be addressed in other ways (and perhaps more cost-effective ways) than by searching widely, with diminishing returns.

### 4. Should a review really have primary and secondary outcomes?

Consistent with good epidemiological practice, systematic reviewers are usually advised to pick a few primary and a few secondary outcomes: for example, it is noted in the Cochrane handbook that

‘*Primary outcomes are the two or three outcomes from among the main outcomes that the review would be likely to be able to address if sufficient studies are identified, in order to reach a conclusion about the effects (beneficial and adverse) of the intervention(s)*’ [18].

Such an approach facilitates hypothesis testing and limits the options for *post hoc* data dredging, thereby increasing the strength of any inferences about causality which are drawn. However, if we accept that systematic reviews do not have to adopt a hypothesis-testing approach (as described above), then this restriction in choice of outcomes to include is not necessary. It may even be at odds with the need for reviews to take a systems-oriented perspective which involves describing the range of impacts of interventions in different settings or contexts. Describing what changes flow from perturbations in a system is one of the main goals of a systems-oriented systematic review. This task may involve focusing on a smaller number of primary and secondary outcomes, but this is not a prerequisite and may even be unhelpful if we are interested in the effects of the intervention across the entire range of outcomes.

The problem of choosing primary outcomes is further compounded when the choice of primary outcome varies according to the stakeholders’ perspectives. Stakeholders often disagree on which outcome is primary or secondary [27]. Designating primary and secondary outcomes is not always helpful in reviews of complex interventions.

### 5. Should reviews really exclude ‘weak’ studies?

‘*It is not helpful to include evidence for which there is a high risk of bias in a review, even if there is no better evidence*.’ The Cochrane handbook, 2011 [18].

There are risks and benefits to decisions about including or excluding weaker evidence. One approach is to reject the highly biased studies. This is easier to implement in fields where there is a plethora of trials and where there is clear agreement about the most important biases and how they affect study conclusions. It is less easy to apply this approach in reviews of complex interventions. One reason is that when a field is in still in development - as is public health intervention research for example - it may be particularly valuable to see the whole range of evidence, and not just the ‘best’ evidence, because even ‘weaker’ studies can provide information of value. For example, they show the range and nature *potential* effects across different subpopulations, which can help with planning further research. More importantly, study quality is often confounded with a range of other study characteristics: ‘high-quality’ and ‘low-quality’ studies do not differ simply in terms of their methodological rigour. In public health, the types of interventions that are evaluated in high-quality studies (for example RCTs) may often be quite different from those that get evaluated in non-experimental studies. In one review of transport interventions to promote cycling and walking, simpler individual level interventions (for example leaflets) were evaluated in RCTs while more upstream interventions (such as improving transport services) were evaluated using observational methods [28,29]. Interventions evaluated in high-quality studies may therefore be systematically different from those that get evaluated in ‘low’-quality studies. This can prevent a systematic reviewer considering an entirely different set of potential policy solutions. This is not an excuse to ignore study quality, but it is a reason to think very carefully about exclusions by study design, or study quality.

### 6. Should qualitative evidence of the impact of interventions be included in systematic reviews of effectiveness?

As noted earlier, systematic reviews of qualitative studies are increasingly common. However, qualitative evidence still has what has sometimes been called a ‘handmaiden,’ or supporting role, in which it is generally used only for hypothesis generation, for elucidating issues around acceptability, and for exploring meanings, experiences and mechanisms, and barriers and facilitators [30]. However, when the question turns to the ‘real’ questions about whether something ‘works’ - rather than ‘how’ it works, then only quantitative studies are believed to provide credible evidence. As a result, reviewers currently exclude qualitative studies from the evidence base on the impacts of interventions (as opposed to processes). In systematic reviews of effectiveness, we do grant qualitative evidence admission, but only to answer supporting questions, such as questions about intervention acceptability.

However, this may undervalue the contribution of qualitative research to understanding intervention *impacts.* Qualitative research can identify the range and nature of impacts of interventions and can give some sense of whether they are rare or common. It can identify unintended, unanticipated impacts. When impacts are large, it may even in principle constitute sufficient evidence that an intervention has caused a particular outcome - for example, when the effect is large, direct and immediate. When we want to *measure* those impacts, then obviously we need quantitative methods - but for identifying whether impacts occur, and to whom and what those are, then qualitative methods also play a crucial part; and in practice, most of the evidence in everyday life on which we base decisions is probably qualitative in nature.

Changing systematic reviewers’ perspective on qualitative evidence involves a move away from current review perspectives. It may require a serious consideration of the types and nature of impacts for which we would accept qualitative evidence; that is, moving qualitative research from a handmaiden role to what Popay has referred to as an ‘enhancement’ role [31]. For example, qualitative data may provide evidence on impacts which have not been measured quantitatively. Some reviews currently do this, but this is uncommon. For example, a recent Campbell Collaboration review presented qualitative evidence that tenants moving from flats to houses feel greater safety [32,33]. Qualitative evidence is sufficient for identifying such an impact; measuring the size of the impact (which may, or may not be useful) however needs additional quantitative data (an ‘enhancement model’ for quantitative data, perhaps). In short, we may be significantly undervaluing what qualitative studies can bring to systematic reviews.

## Discussion

These issues are not all easily addressed, nor do they need to be. They are not proposals; instead, they are a list of challenges that reviewers may need to think through further as review methods continue to develop. The challenges are particularly likely to apply to complex reviews where ‘hard and fast’ rules do not always result in useful reviews.

They are not addressed particularly to reviewers in the Cochrane or Campbell Collaborations, as both collaborations are increasingly broad churches, and many reviewers in any case are moving away from simple reviews of effect sizes. The target is the large corpus of reviews (and also funders) inside and outside Cochrane which use systematic review methods in a cookbook fashion. However complex the intervention, the goal of such reviews too often simply appears to be to find as many effect sizes as possible.

The above list of challenges was selected for discussion because they relate to some of the basic principles and practices of systematic reviews. However, what links them is that once reviewers move away from the focused testing of hypotheses about effectiveness and move away from asking ‘what works’ to ‘what happens,’ then it becomes inevitable that many core systematic review practices need to change, starting with the systematic review question. Narrow scientific questions to complex questions are attractive, but they carry the large risk of producing findings with little value for real-life decision-makers.

A perhaps simpler way of expressing the above arguments is to simply state that the current model of systematic reviews is mainly a frequentist one, which automatically leads reviewers into searching for as much ‘gold standard’ evidence as possible to test suitable hypotheses. This may be why reviews, particularly reviews of complex interventions, often fail to produce much more than the conclusion that the evidence is ‘weak’ or ‘mixed’ and struggle to incorporate and integrate different types of evidence - such as different designs and evidence from different contexts. The information value of such reviews is low. We usually already know before the review starts that the evidence is likely to be ‘weak’, or ‘mixed’, because complex phenomena are difficult to evaluate, and so ‘hard tests’ of hypotheses are uncommon.

By comparison, a Bayesian perspective in which the purpose of systematic reviews is to assemble evidence - of different types, if necessary - in order to inform decisions (rather than to test hypotheses) would seem much more promising and productive. In such a perspective, integration of evidence from different designs and contexts is much less problematic; better use is made of all available data, and each new piece of evidence potentially contributes to a decision. The ‘what happens’ question outlined above falls into this category: The decision maker uses a wide range of evidence to help consider ‘what has happened’ previously when ‘this’ intervention has been implemented in different settings and under different conditions and uses this to make an informed judgement about whether to implement it in a new setting. In effect, this is what some reviews of complex interventions actually do at present, sometimes implicitly, but the reviews are presented, conducted, analysed and reported as if they were testing hypotheses. Openly accepting that evidence synthesis often is, and should be, an exercise in Bayesian decisionmaking, and reducing uncertainty, and not hypothesis testing, is overdue.

Finally, it is also worth briefly considering whether there are core systematic review principles that are ‘*non*-negotiable’. The main one of these must be the transparency of methods. The value of transparency is that anyone can see and challenge how evidence is selected, weighed and synthesised. The Royal Statistical Society motto ‘*Nulla in verba*’ (‘take nobody’s word for it’) can be applied to systematic reviewers just as much as to other experts.

## Conclusion

While systematic reviews are being applied to ever more complex questions, even when they do so, they are still often driven by quite a simple hypothesis-testing epistemology, searching for every possible study (whether or not this is efficient, or necessary) and excluding ‘weak’ studies (with insufficient consideration of the hidden biases this may introduce). This approach is often appropriate, particularly when there is a strong evidence base (a lot of trials) and when simpler answers are needed. Very often it is not appropriate, and simplistic reviews may reach simplistic conclusions. There is a rich research agenda here for future methodological research which should help make systematic reviews more efficient and meaningful, though to do so we may sometimes need to abandon or significantly amend some aspects of current practice and thinking. Among areas for potentially useful future work include the examination of publication bias in qualitative research; research on the efficiency and potential biases of comprehensive searches in different disciplines; the use of Bayesian methods in synthesis (as in the example from Roberts and colleagues in 2002) [34], which place the synthesis in a decision-making framework rather than a hypothesis-testing framework [35]; and perhaps most pressingly, the development of a systems approach (as opposed to a complex interventions approach) to systematic reviews.

## References

[1] Glass G. Meta-Analysis at 25. Available online at: http://www.gvglass.info/papers/meta25.html Accessed 11th August 2014.

[2] Chandler J, Hopewell S (2013). Cochrane methods–twenty years experience in developing systematic review methods. Syst Rev.

[3] Schünemann H, Tugwell P, Reeves B, Akl EA, Santesso N, Spencer FA (2013). Non-randomized studies as a source of complementary, sequential or replacement evidence for randomized controlled trials in systematic reviews on the effects of interventions. Res Syn Meth..

[4] Petticrew M, Roberts H (2006). Systematic reviews in the social sciences.

[5] Whelan J, Love P, Pettman T, Doyle J, Booth S, Smith E (2014). Cochrane update: predicting sustainability of intervention effects in public health evidence: identifying key elements to provide guidance. J Public Health (Oxf).

[6] Thomas J, Harden A, Oakley A, Oliver S, Sutcliffe K, Rees R (2004). Integrating qualitative research with trials in systematic reviews. Br Med J.

[7] Dixon-Woods M, Agarwal S, Young B, JOnes D, Sutton A (2004). Integrative approaches to qualitative and quantitative evidence.

[8] Campbell R, Britten N, Pound P, Donovan J, Morgan M, Pill P (2006). Using meta-ethnography to synthesise qualitative research, Moving beyond effectiveness in evidence synthesis.

[9] Popay J, Roberts H, Sowden A, Petticrew M, Arai L, Rodgers M. Guidance on the conduct of narrative synthesis in systematic review: ESRC. 2006. Available at: http://www.lancs.ac.uk/shm/research/nssr/research/dissemination/publications/NS_Synthesis_Guidance_v1.pdf. Accessed on 6.7.2012, 2006.

[10] Greenhalgh T, Wong G, Westhorp G, Pawson R (2011). Protocol - realist and meta-narrative evidence synthesis: evolving standards (RAMESES). BMC Med Res Methodol.

[11] Wong G, Greenhalgh T, Westhorp G, Buckingham J, Pawson R (2013). RAMESES publication standards: meta-narrative reviews. BMC Med.

[12] Wong G, Greenhalgh T, Westhorp G, Buckingham J, Pawson R. RAMESES publication standards: realist syntheses. BMC Med. 2013;11(21) doi:10.1186/1741-7015-11-21. 10.1186/1741-7015-11-21 PMC3558331 23360677

[13] Hannes K, Booth A, Harris J, Noyes J (2013). Celebrating methodological challenges and changes: reflecting on the emergence and importance of the role of qualitative evidence in Cochrane reviews. Syst Rev.

[14] Sackett D, Haynes R, Guyatt G, Tugwell P (1991). Clinical epidemiology.

[15] Mulrow CD (1994). Rationale for systematic reviews. Br Med J.

[16] Gough D, Oliver S, Thomas J (2012). An introduction to systematic reviews.

[17] Sandelowski M, Voils C, Leeman J, Crandell JL (2012). Mapping the mixed methods–mixed research synthesis terrain. J Mix Methods Res.

[18] Higgins J, Green S, editors. Cochrane handbook for systematic reviews of interventions Version 5.1.0 [updated March 2011]: The Cochrane Collaboration. 2011. Available from www.cochrane-handbook.org., 2011.

[19] Moher D (2013). The problem of duplicate systematic reviews. Br Med J.

[20] Booth A, Clarke M, Dooley G, Ghersi D, Moher D, Petticrew M (2013). PROSPERO at one year: an evaluation of its utility. Syst Rev.

[21] Hawe P, Shiell A, Riley T (2009). Theorising interventions as events in systems. Am J Community Psychol.

[22] Shea B, Grimshaw J, Wells G, Boers M, Andersson N, Hamel C (2007). Development of AMSTAR: a measurement tool to assess the methodological quality of systematic reviews. BMC Med Res Methodol.

[23] Lorenc T, Pearson R, Jamal F, Cooper C, Garside R (2012). The role of systematic reviews of qualitative evidence in evaluating interventions: a case study. Res Synth Methods.

[24] Claxton K, Sculpher M (2006). Using value of information analysis to prioritise health research - some lessons from recent UK experience. Pharmacoeconomics.

[25] Booth A (2010). How much searching is enough? Comprehensive versus optimal retrieval for technology assessments. Int J Technol Assess Health Care.

[26] Egger M, Jüni P, Bartlett C, Holenstein F, Sterne J (2003). How important are comprehensive literature searches and the assessment of trial quality in systematic reviews?. Health Technol Assess.

[27] Petticrew M (2013). Plural evidence in public health evaluation: epistemological challenges to evidence production and use. Evid Policy.

[28] Ogilvie D, Egan M, Hamilton V, Petticrew M (2004). Promoting walking and cycling as an alternative to using cars: what works? A systematic review. Br Med J.

[29] Ogilvie D, Egan M, Hamilton V, Petticrew M (2005). Systematic reviews of health effects of social interventions: 2. Best available evidence: how low should you go?. J Epidemiol Community Health.

[30] Popay J (2007). Plenary presentation.

[31] Popay J (2003). Qualitative research and the epidemiological imagination: a vital relationship. Gac Sanit.

[32] Gibson M, Thomson H, Kearns A, Petticrew M (2011). Understanding the psychosocial impacts of housing type: qualitative evidence from a housing and regeneration intervention. Hous Stud.

[33] Thomson H, Thomas S, Sellström E, Petticrew M. Housing improvements for health and associated socio-economic outcomes: a systematic review. Campbell Syst Rev. 2013. p. 2. doi: 104073/csr20132. 10.1002/14651858.CD008657.pub2 PMC12551615 23450585

[34] Roberts K, Dixon-Woods M, Fitzpatrick R, Abrams KR, Jones DR (2002). Factors affecting uptake of childhood immunisation: a Bayesian synthesis of qualitative and quantitative evidence. Lancet.

[35] Threlfall AG, Meah S, Fischer AJ, Cookson R, Rutter H, Kelly MP (2015). The appraisal of public health interventions: the use of theory. J Public Health.

